# Hormonal Balance in Relation to Expression of Selected Genes Connected with Hormone Biosynthesis and Signalling—The Effect of Deacclimation Process in Oilseed Rape

**DOI:** 10.3390/ijms26157408

**Published:** 2025-08-01

**Authors:** Magdalena Rys, Jan Bocianowski, Michał Dziurka, Barbara Jurczyk, Julia Stachurska, Piotr Waligórski, Anna Janeczko

**Affiliations:** 1 The Franciszek Górski Institute of Plant Physiology, Polish Academy of Sciences, Niezapominajek 21, 30-239 Krakow, Poland; michal.dziurka@gmail.com (M.D.); jstachurska@ifr-pan.edu.pl (J.S.); p.waligorski@ifr-pan.edu.pl (P.W.); a.janeczko@ifr-pan.edu.pl (A.J.); 2 Department of Mathematical and Statistical Methods, Poznań University of Life Sciences, Wojska Polskiego 28, 60-637 Poznań, Poland; jan.bocianowski@up.poznan.pl; 3 Department of Plant Breeding, Physiology and Seed Science, Faculty of Agriculture and Economics, University of Agriculture in Kraków, Podłużna 3, 30-239 Krakow, Poland; barbara.jurczyk@urk.edu.pl

**Keywords:** auxins, *Brassica napus*, cold acclimation, cytokinins, deacclimation, dehardening, gibberellins, oilseed rape, stress hormones

## Abstract

Global climate change is causing increasing fluctuations in winter temperatures, including episodes of warm conditions above 9 °C. Such events disrupt cold acclimation in plants and can induce deacclimation, reducing frost tolerance and altering, among other things, hormonal regulation. This study investigated hormonal and molecular changes associated with cold acclimation and deacclimation in oilseed rape (*Brassica napus* L.) cultivars Kuga and Thure. Plants were grown under different conditions: non-acclimated (17 °C for three weeks), cold-acclimated (4 °C for three weeks), and deacclimated (16/9 °C day/night for one week). Detailed hormone analysis included auxins, gibberellins, cytokinins, stress-related hormones, and the expression of hormone-related genes (*BnABF2*, *BnAOS*, *BnARF1*, *BnARR6*, *BnICS1*, *BnRGA*, and *BnWRKY57*). Hormone concentrations in leaves changed dynamically in response to deacclimation with increased amounts of growth-promoting hormones and decreased amounts of stress hormones. Additionally, alterations in gene expression during deacclimation, such as in *BnABF2* and *BnICS1*, may function as protective mechanisms to help maintain or regain frost tolerance during reacclimation when temperatures decline again after the warm period. These findings improve the understanding of hormonal and molecular responses involved in the deacclimation of oilseed rape.

## 1. Introduction

Oilseed rape (*Brassica napus* spp. oleifera L.) is a plant from the *Brassicaceae* family and one of the most significant oil plants in the world after oil palm and soybean. The area cultivated with oilseed rape is constantly growing, and in 2023, it was about 35 million hectares throughout the world and almost 12 million hectares in Europe. The obtained world yield was about 87 million tons, and the European one about 30 million tons, of which over 19 million tons fell in the European Union. In Poland, where it is one of the most commonly cultivated winter crops, almost 3.5 million tons of oilseed rape are produced annually [Food and Agriculture Organization of the United Nations. Data available online: https://www.fao.org/faostat/en/#data/QCL/visualize (accessed on 20 March 2025)].

As the global human population grows, and it is expected to continue to grow, oilseed rape plays a key role in meeting the global demand for edible vegetable oil, as it has a favourable fatty acid composition and a well-balanced amino acid content.

Oilseed rape is also used as an ingredient in animal feed and for biodiesel production [1]. Additionally, it contributes to sustainable agriculture by improving soil structure, as it is used as an effective pre-crop in crop rotations [2]. Due to its yellow, melliferous flowers, it is an important plant for pollinators.

The winter cultivars of oilseed rape are more often cultivated in many countries than the spring cultivars, because they produce higher yields. However, their growth occurs during the wintertime, which exposes them to frost injuries, which can lead to economic losses. However, such species have developed mechanisms that allow them to survive at temperatures below 0 °C.

The first stage in acquiring frost resistance is pre-hardening, which takes place at temperatures such as +12 °C to +7 °C. Then comes the hardening process—cold acclimation (cold hardening). It is a low temperature (usually +1 to +5 °C)-induced process, which lasts for about a few weeks in the autumn and causes a significant increase in frost tolerance and many physiological, biochemical, and molecular changes. Those are, among others, changes in the lipid components of the membranes [3,4], content and composition of carbohydrates and other osmolytes (proline, betaine, etc.) [5,6], decrease in water content in tissues and increased osmotic potential [7], increased content of stress hormones, e.g., ABA [8], an increased accumulation of protective proteins, e.g., heat shock proteins [9,10], and the strengthening of the antioxidant system [11,12].

Unfortunately, due to climate changes in recent years, periods of warm breaks with higher temperatures (over 9 °C, sometimes reaching even 20 °C) are becoming more frequent in winter, lasting for a few days, which interrupt and disturb the natural processes of acclimation to cold in winter crops and make plants more susceptible to frost [13,14,15,16]. This phenomenon is called deacclimation (dehardening), and its rate is closely related to the values of higher temperatures, its duration, as well as the species and genotype of the plant [17]. It can cause yield losses resulting from crop damage caused by frost following a period of warm break. Despite the increasing number of studies devoted to the detailed physiological, biochemical, and molecular changes accompanying the deacclimation process, knowledge about them is still limited. In our research to date, we have demonstrated the changes that occur in the oilseed rape metabolism as a result of the deacclimation process. Our previous studies have shown that a one-week break at 16 °C/9 °C (d/n) reverses the cold acclimation effect, and many biochemical/physiological parameters return to values similar to those observed in the non-acclimated control. Additionally, deacclimation caused a decrease in frost tolerance in comparison to cold-acclimated plants [7,14], which may result in stem elongation or even the development of buds. As a result of deacclimation, there was an increase in the efficiency of both the light and dark reactions of photosynthesis, a decrease in sugar content, and a rise in osmotic potential [7]. It was also well correlated with increasing leaf relative water content and a decrease in the accumulation of the aquaporin protein BnPIP1 [7,14]. Moreover, deacclimation was also shown to cause a decrease in the accumulation of protective proteins from a group of heat shock proteins (HSP), from the WCS120 protein family [18], and influence phytohormonal homeostasis [10]. Additionally, the deacclimation process changes the content of antioxidants [19] and pattern of gene expressions related to the cold response [20].

A plant’s metabolism is controlled by phytohormones (gibberellins—GAs, auxins—AUXs, and cytokinins—CKs and so-called stress hormones such as: abscisic acid—ABA, jasmonic acid—JA, salicylic acid—SA, and ethylene—ET, as well as brassinosteroids—BR), so individual and complex interactions of phytohormones play an important role in the process of plant adaptation to abiotic stress conditions through their biosynthesis, transport, and signalling pathways [8,21]. Plant hormones participate in regulating the physiological and metabolic processes necessary for plants to acquire cold tolerance and survive at low temperatures.

Gibberellins (GAs) are growth-promoting hormones that typically decrease during cold acclimation, which helps to inhibit growth and allows the plant to focus on developing cold tolerance mechanisms [8,22]. The *RGA* gene encodes the DELLA protein, which acts as a repressor in the gibberellin signalling pathway, influencing various aspects of plant growth and development. The direct involvement of *RGA* gene expression to cold acclimation processes is not yet fully elucidated and remains a subject of research.

Auxins (AUXs) are the phytohormones that regulate various aspects of plant growth and development, such as cell elongation, organ development, and tropisms [23]. Auxins also play a role in plant responses to various stresses and interact with other hormones to enhance stress tolerance. Under cold stress, levels of auxins change differently depending on plant species, developmental stage, or various physiological aspects [24,25,26,27]. Auxins regulate many genes, and the *auxin response factors* (*ARFs*) are genes that play a vital role in auxin signalling and regulating the expression of auxin-responsive genes [28]. *ARFs* are also involved in plant responses to environmental stresses [29,30,31].

Cytokinins (CKs) are primarily associated with cell division and growth regulation [32]. Under abiotic stress conditions, CKs regulate physiological processes by improving photosynthetic efficiency, enhancing antioxidant enzyme activity, and optimizing root architecture [33]. Their response varies with stress intensity. In extreme drought, CTK signalling and synthesis are inhibited [34], whereas moderate stress promotes survival through typical biological activities [35]. *ARR* genes, which are regulators of CK signalling, play an important role in the plant’s cold response. However, direct measurements of the amounts of different forms of cytokinins showed that low temperatures did not significantly change cytokinin levels, whereas the expression of *ARR* genes significantly increased during cold stress [36]. ARR proteins regulate hormone effects as both positive and negative regulators. Type-B ARRs, activated by cytokinin, promote cytokinin-responsive genes, including Type-A ARR genes. In contrast, Type-A ARRs are quickly upregulated by cytokinin and act as negative regulators, reducing the cytokinin signal [37].

Abscisic acid (ABA) plays a key role in cold acclimation. Its levels increase in response to low temperatures, acting as a signalling molecule [38]. ABA also mediates the accumulation of osmoprotectants such as proline and soluble sugars, which contributes to the osmotic balance of cells under abiotic stress [39]. ABA accumulation activates transcription factors such as CBF (C-repeat binding factors), which increase the expression of cold-responsive genes (CORs) via an ABA-dependent signalling pathway [40]. ABA-responsive element binding factors (AREB/ABF) are transcription factors regulating the expression of ABA-related genes that encode proteins that increase the ability of plants to withstand freezing temperatures by stabilizing cellular structures and protecting them from dehydration and oxidative damage [41].

Salicylic acid (SA) is a simple phenolic compound, which plays an important signalling role in plants under various stress conditions [42,43,44]. SA also participates in plant defence from stresses through crosstalk with other hormones [45,46]. SA signalling leads to promotion of the activation of the antioxidant system, preservation of cell membrane stability, regulation of gene expression and protein synthesis, and promotion of low-temperature signal production [43]. With SA synthesis, the *isochorismate synthase 1* (*ICS1*) gene is associated, because it encodes a key enzyme in SA production. Thus, the expression of *ICS1* plays an important role in plant immunity by regulating the concentration of SA [47].

Jasmonic acid (JA) is a plant hormone involved in regulating various physiological processes such as the development of roots and stems, leaf senescence, and mediating responses to environmental stresses [48,49]. JA also functions as a signalling molecule that modulates the expression of stress-responsive genes in response to abiotic stress conditions. Under low-temperature conditions, the expressions of JA synthesis-related genes, including *allene oxide synthase 1* (*AOS1*), are induced, which leads to increases in endogenous JA levels [50,51].

This work is a continuation of the research on the deacclimation process of oilseed rape conducted in our group. As we have shown in the previous paper [10], deacclimation causes serious disruptions in phytohormonal balance; therefore, we decided to investigate changes in the expression of selected genes related to hormone biosynthesis and signalling.

## 2. Results

### 2.1. Phytohormones in Leaves

**Figure 1:**
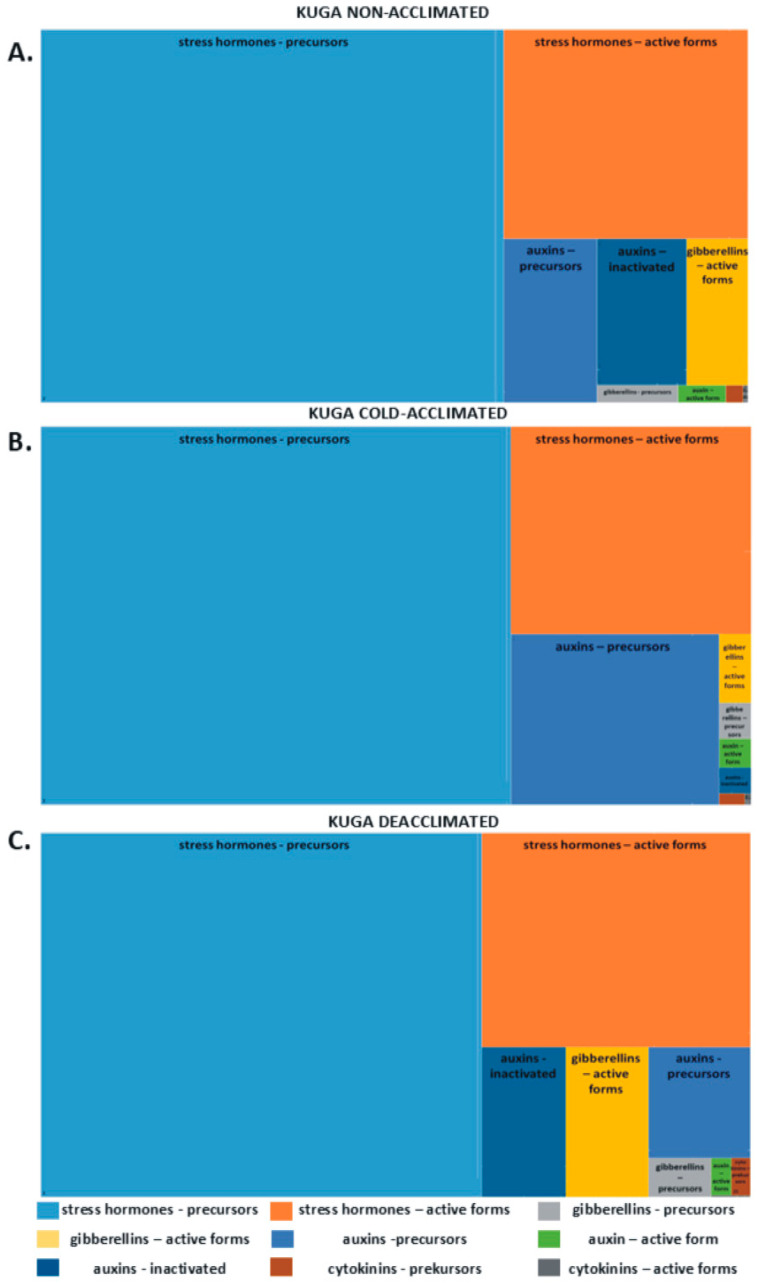

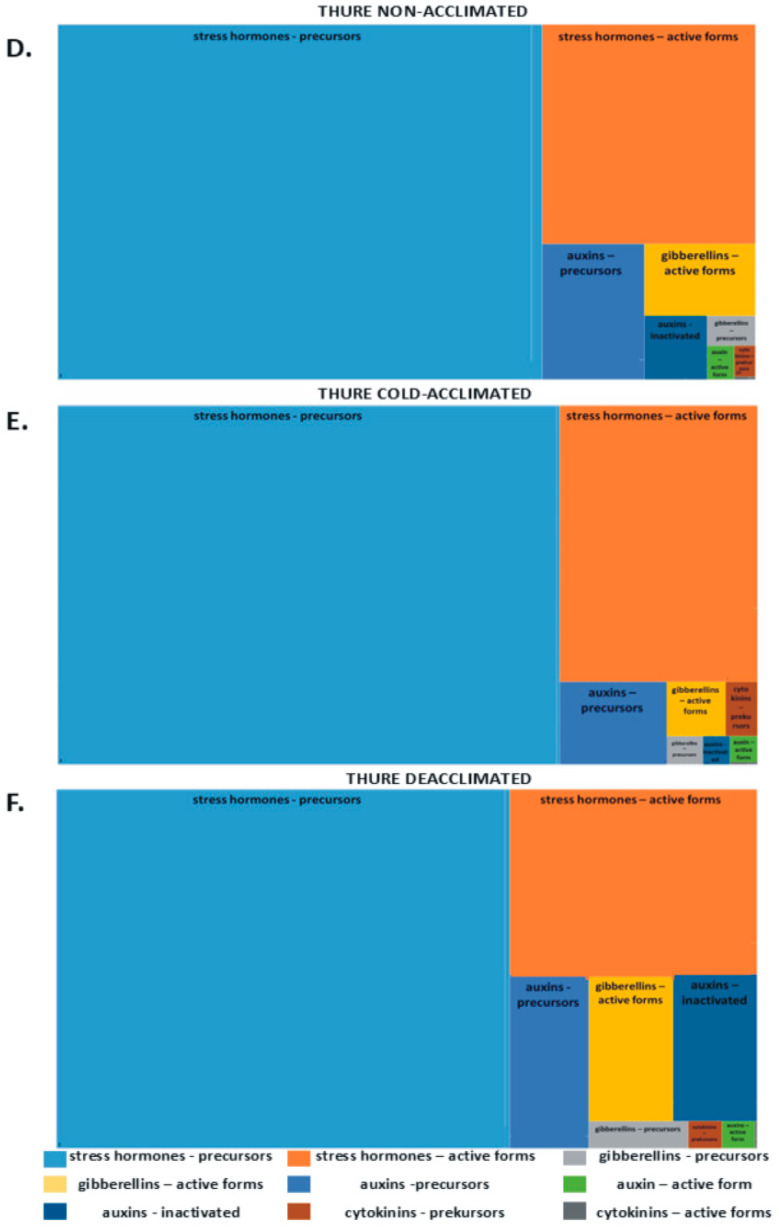
Hierarchical tree diagram with the percentage content of groups of phytohormones: auxins, cytokinins, gibberellins, and stress hormones in the total pool of hormones in cv. Kuga: (**A**) non-acclimated plants, (**B**) cold-acclimated plants, and (**C**) deacclimated plants and in cv. Thure: (**D**) non-acclimated plants, (**E**) cold-acclimated plants, and (**F**) deacclimated plants.

The hierarchical tree diagram shows the percentage content of groups of phytohormones: auxins, cytokinins, gibberellins, and so-called stress hormones (abscisic, jasmonic, and salicylic acids) in the total pool (Figure 1). In both tested cultivars, stress hormone precursors represented the highest amount of all hormones. Non-acclimated and deacclimated plants, in comparison to cold-acclimated ones, are characterized by greater amounts of growth-promoting hormones (e.g., active forms of gibberellins and their precursors). Cold-acclimated plants exhibit a greater accumulation of active forms of stress hormones (Figure 1A–F).

So-called stress hormones such as abscisic acid (ABA), jasmonic acid (JA), and salicylic acid (SA) and their precursors 12-oxo-phytodenoic acid (12-oxo-PDA) and benzoic acid (BeA), were identified and shown in Table 1.

In cold-acclimated plants, was observed a much lower content of 12-oxo-phytodenoic acid (12-oxo-PDA), which is a precursor of jasmonic acid, in comparison to non-acclimated plants. In cv. Kuga, it was more than 2-fold, and in cv. Thure, it was almost a 4-fold decline. After deacclimation, the 12-oxo PDA content increased 2-fold in deacclimated cv. Thure plants, and in the case of cv. Kuga, there were no changes between the CA and DA plants.

A similar tendency in changes was recorded for the benzoic acid (BeA), which is a precursor of salicylic acid. Lower amounts of BeA were detected in leaves of both cultivars after cold acclimation compared to the non-acclimated plants. Deacclimation reversed this process, but all the changes were statistically insignificant.

The concentration of abscisic acid (ABA) increased over 2-fold in the cold-acclimated plants of both tested cultivars compared to non-acclimated plants. After the deacclimation process, the effects of the cold were generally reversed, and the content of ABA decreased by almost 32% in cv. Kuga leaves and 19% in cv. Thure leaves.

No changes in the content of jasmonic acid (JA) were observed between the NA and CA cv. Kuga plants. Cold acclimation caused a more than 2.5-fold higher accumulation of JA in the leaves of cv. Thure compared to NA plants. Deacclimation reduced the content of JA in cv. Thure plants by almost 20%, but in cv. Kuga DA plants, there was over 6-fold higher content of JA than in CA plants. A decrease in the concentration of salicylic acid (SA) in the cold-acclimated plants was noted by 24% in cv. Kuga and by 10% in cv. Thure, respectively. In both cultivars, there were no statistically significant differences in the SA concentration between the CA and DA plants.

In our studies, the following gibberellins, divided into precursors and active forms were identified: GA15, GA9, GA53, GA44, GA19, GA20—precursors and GA4, GA7, GA3, GA6, GA1, GA5—active forms and shown in Table 1.

Cold acclimation generally decreased the levels of all gibberellin precursors in both tested cultivars, with levels increasing again after deacclimation.

GA15 decreased by 13% in cv. Kuga and 53% in cv. Thure during cold acclimation. After deacclimation, only cv. Thure showed an increase in GA15, while cv. Kuga continued to decline, though these changes were not statistically significant.

GA9 levels were approximately 30% lower in cv. Kuga and 20% lower in cv. Thure compared to non-acclimated plants but increased significantly after deacclimation—by 19% in cv. Kuga and 31% in cv. Thure.

For GA53, cold acclimation reduced levels by over 62% in cv. Kuga and 24% in cv. Thure. After deacclimation, GA53 levels remained low in cv. Kuga but approached those of non-acclimated cv. Thure.

GA44 levels slightly decreased with cold acclimation but returned to similar levels in both non-acclimated and deacclimated plants. GA19 dropped by 15% in cv. Kuga and 54% in cv. Thure, but after deacclimation, rose nearly 7-fold in cv. Kuga and over 2.5-fold in cv. Thure.

There were no significant changes in GA20 in cv. Kuga, while cv. Thure saw GA20 increase over 12.6-fold after deacclimation. GA4 levels decreased in both cultivars after cold acclimation but returned to normal post-deacclimation.

GA7 levels fell significantly in both cultivars due to cold acclimation—68% in cv. Kuga and 36% in cv. Thure—but increased substantially after deacclimation. GA3 decreased by 87% in cv. Kuga and 68% in cv. Thure, with levels increasing over 11-fold in cv. Kuga and almost 6-fold in cv. Thure after deacclimation.

GA6 levels dropped by over 63% during cold acclimation but increased in both cultivars after deacclimation. There were no significant differences in GA1 between the different treatments, although a slight decrease was observed in both cultivars after cold acclimation.

In cv. Kuga, GA5 levels remained similar to non-acclimated plants, while cv. Thure saw a 42% increase compared to non-acclimated plants. Deacclimation resulted in an 18% increase in cv. Kuga and a nearly 5-fold increase in cv. Thure.

The study identified several auxin precursors, including indole-3-acetamide (IAM) and indole-3-acetonitrile (IAN), along with the active auxin indole-3-acetic acid (IAA) and metabolites such as indole-3-acetyl-aspartic acid (IAAsp), oxoindole-3-acetic acid (OxIAA), indole-3-acetyl-glutamic acid (IAA-Glu), and indole-3-carboxylic acid (I3CA), as shown in Table 1.

In cold-acclimated cv. Kuga plants, IAM levels increased by 3.8-fold compared to non-acclimated (NA) plants but decreased to NA levels after deacclimation. In contrast, cv. Thure plants showed a more than 3-fold decrease in IAM after cold acclimation. The IAN content in cv. Kuga was nearly 78% higher after cold acclimation but decreased by over 57% post-deacclimation. Conversely, IAN levels in cv. Thure dropped by almost 35% during acclimation, with recovery during deacclimation.

Cold acclimation reduced IAA concentrations significantly in both cultivars, with cv. Kuga showing no change during deacclimation, while cv. Thure experienced a 46% increase. IAAsp levels decreased by over 3.5-fold during cold acclimation but returned to NA levels after deacclimation.

IAA-Glu levels were lower in cold-acclimated plants, but after deacclimation, cv. Kuga saw a 4.8-fold increase and cv. Thure a 2.4-fold increase. There were no differences in OxIAA between NA, cold-acclimated (CA), and deacclimated (DA) plants in cv. Kuga, while cv. Thure showed a significant decrease of over 30%.

I3CA levels dropped to zero in both cultivars after cold acclimation but returned to NA levels in cv. Kuga after deacclimation and increased nearly 5-fold in cv. Thure.

Several key cytokinins were identified, including isopentenyl adenosine (IPA), trans-zeatin (t-ZEA), cis-zeatin (c-ZEA), trans-zeatin riboside (t-ZEA-R), and cis-zeatin riboside (c-ZEA-R), as shown in Table 1.

In the Kuga cultivar, IPA decreased by 20% after cold acclimation but increased nearly 3-fold after deacclimation. In contrast, the Thure cultivar showed a 2.5-fold increase in IPA after cold acclimation, with a significant decrease upon deacclimation.

Both cold acclimation and deacclimation caused a notable decrease in t-ZEA levels. Kuga plants experienced over an 80% reduction in t-ZEA after acclimation and over 93% after deacclimation. The Thure cultivar showed no statistically significant changes.

Cis-zeatin levels increased slightly during cold acclimation, with further increases post-deacclimation (over 53% for Kuga and 132% for Thure). Cold acclimation also raised t-ZEA-R levels by over 40% in Kuga and 15% in Thure, although a slight reduction occurred during deacclimation. The changes in c-ZEA-R levels were minor and not statistically significant.

Overall, cold acclimation decreased total active forms of cytokinins, with variations observed between cultivars.

### 2.2. Phytohormones in Cell Sap

**Figure 2:**
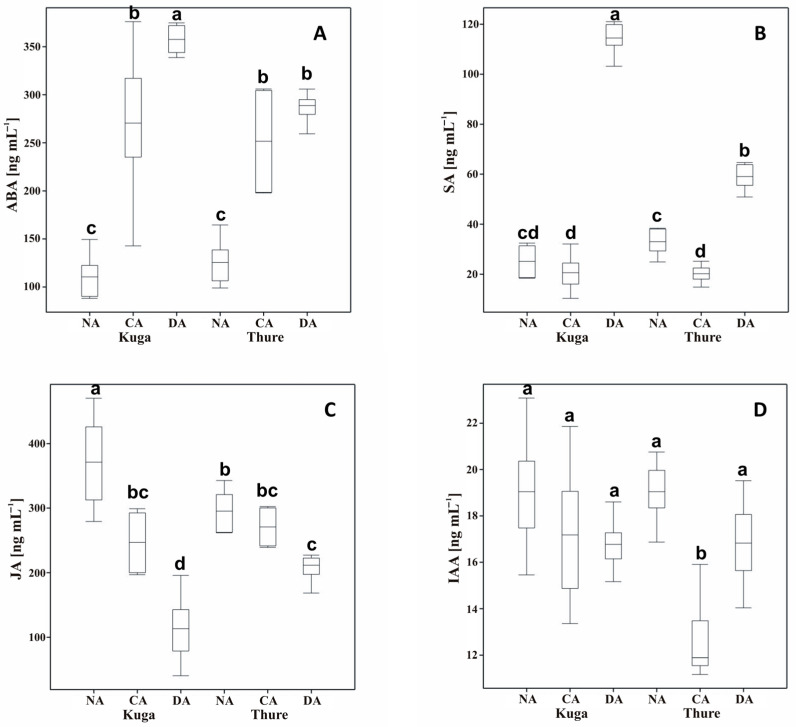
Content of the stress hormones: abscisic acid (ABA—(**A**)), salicylic acid (SA—(**B**)), jasmonic acid (JA—(**C**)), and active auxin indole-3-acetic acid (IAA—(**D**)) in cell sap obtained from leaves of two cultivars (Kuga and Thure) of the non-acclimated (NA), cold-acclimated (CA), and deacclimated (DA) oilseed rape. Values marked with the same letters were not significantly different according to the Duncan test (*p* ≤ 0.05).

The concentration of abscisic acid (ABA) in cell sap increased in all of the cold-acclimated plants compared to the non-acclimated plants by almost 145% in cv. Kuga and 100% in cv. Thure. In the deacclimated plants, there was still a significantly higher content of ABA than in the CA plants. There was an increase of 32% for cv. Kuga and 13% for cv. Thure (Figure 2A).

Cold treatment caused a decrease in the level of salicylic acid (SA) in both cultivars—over 18% for cv. Kuga and almost 39% for cv. Thure in comparison to non-acclimated plants. As a result of the deacclimation process, there was a statistically significant increase in the content of SA of over 6-fold for cv. Kuga and almost 3-fold for cv. Thure when compared to CA plants (Figure 2B).

A lower amount of jasmonic acid (JA) was observed in both cultivars of cold-acclimated plants—over 33% for cv. Kuga and over 8% for cv. Thure compared to the NA plants. After the deacclimation process, even lower values were noted. And so, for the cv. Kuga, it was over 54%, and for the cv. Thure, it was over 23% lower, respectively (Figure 2C).

The content of the active auxin indole-3-acetic acid (IAA) decreased after cold acclimation, but only for the Thure cultivar was the difference statistically significant (by over 33% in comparison to NA plants). The content of IAA after deacclimation was dependent on the cultivar. For the Kuga cultivar, there was a slight decrease (about 2%) compared to the CA plants, and for the Thure cultivar, there was a statistically significant increase by over 33% (Figure 2D).

### 2.3. Expression of the BnRGA, BnARF1, BnARR6, BnABF2, BnISC1, BnAOS, and BnWRKY57

**Figure 3:**
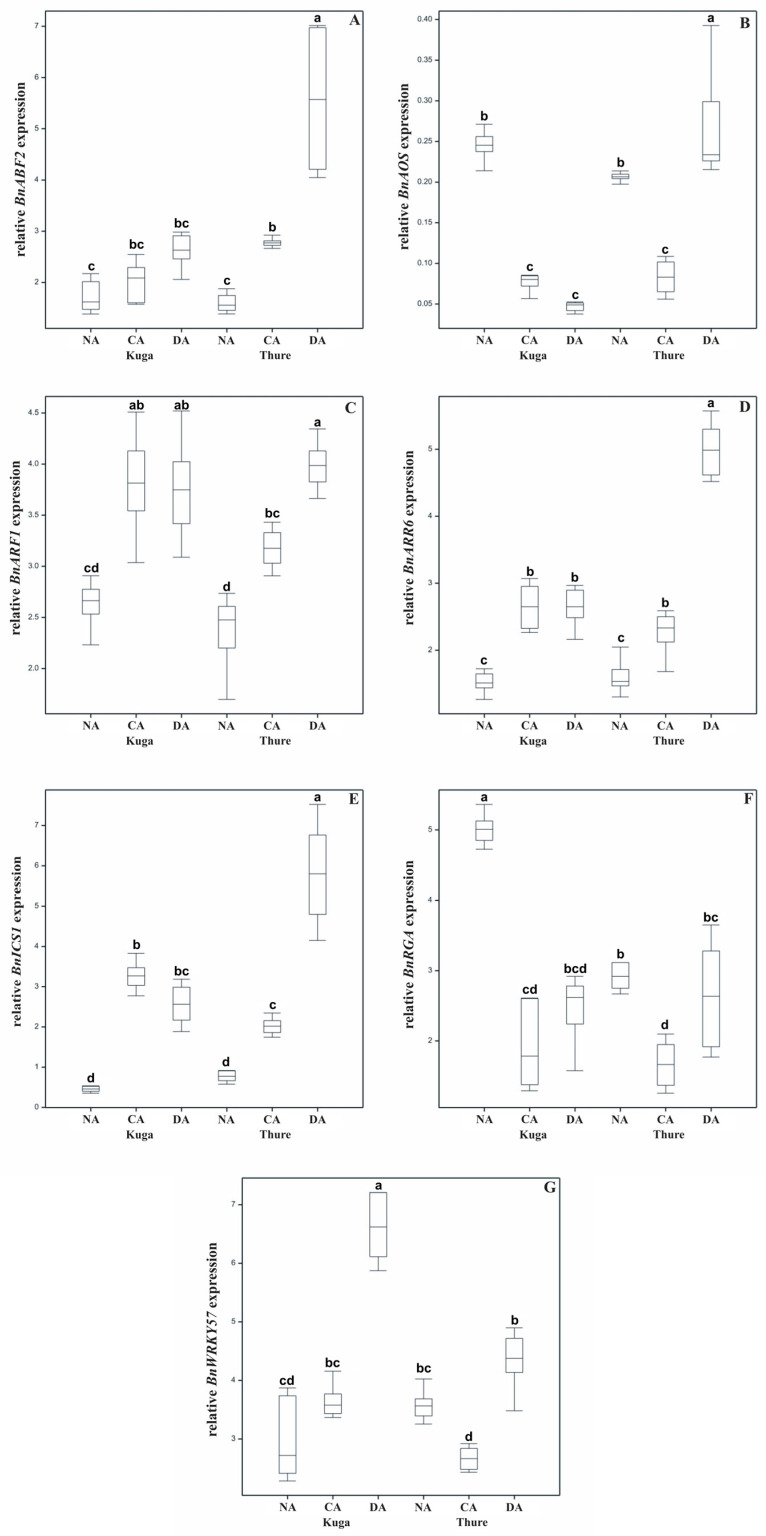
Accumulation of the hormone-related transcript genes—*BnABF2* (**A**), *BnAOS* (**B**), *BnARF1* (**C**), *BnARR6* (**D**), *BnICS1* (**E**), *BnRGA* (**F**), and *BnWRKY57* (**G**) in the leaves of the non-acclimated (NA), cold-acclimated (CA), and deacclimated (DA) oilseed rape cv. Kuga and cv. Thure. The results of the accumulation of the transcripts are presented as the fold change in the expression of the *BnABF2*, *BnAOS*, *BnARF1*, *BnARR6*, *BnICS1*, *BnRGA*, and *BnWRKY57* genes in the given samples compared to the endogenous reference gene *actin*. Values marked with the same letters were not significantly different according to the Duncan test (*p* ≤ 0.05).

The accumulation of *BnABF2* transcript levels increased in both cultivars due to the cold acclimation; next, it increased even further after deacclimation—statistically significant for cv. Thure when compared to CA plants (Figure 3A).

The expression of the *BnAOS* significantly decreased after cold acclimation in both tested cultivars. After deacclimation, the accumulation of this transcript decreased once again in the cv. Kuga plants and increased significantly in cv. Thure deacclimated plants (Figure 3B).

The lowest *BnARF1* transcript levels were noted in the non-acclimated plants. The cold-acclimated plants were characterized by an increased level of this transcript level, and for deacclimated plants, the *BnARF1* transcript level was at a similar level for cv. Kuga and the highest for cv. Thure (Figure 3C).

The level of *BnARR6* increased in the cold-acclimated plants of both cultivars compered to non-acclimated plants. In the cv. Kuga plants, expression of *BnARR6* was maintained after deacclimation, and in the cv. Thure cultivar, it increased by almost 2-fold (Figure 3D).

An increase in the *BnICS1* transcript level was observed in the cold-acclimated plants of both cultivars. Deacclimation reduced the *BnICS1* transcript level in cv. Kuga plants compared to CA plants. There was an opposite effect for the deacclimated plants of cv. Thure, in which a significantly higher expression of *BnICS1* was observed (Figure 3E).

Cold acclimation significantly lowered the accumulation of the *BnRGA* transcript level in both tested cultivars in comparison to non-acclimated plants. After the deacclimation process, the effects of the cold were reversed in both cultivars (Figure 3F).

In cold-acclimated cv. Kuga plants, a small increase in the expression of *BnWRKY57* was observed and a decrease in cv. Thure plants. After deacclimation, the *BnWRKY57* transcript level significantly increased in both cultivars (Figure 3G).

### 2.4. Correlations Between Hormone Concentrations in the Leaves, in the Cell Sap, and the Relative Expression of Hormone-Related Transcript Genes

**Figure 4:**
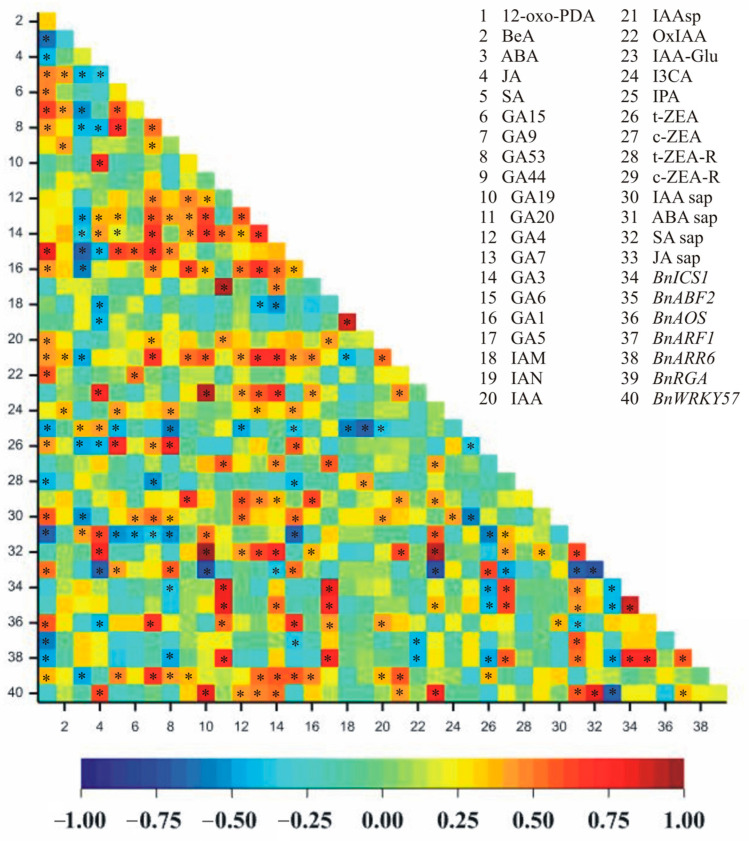
Heatmaps presenting data for correlation coefficients calculated for the accumulation of hormones in leaves and cell sap, and the gene expressions of oilseed rape plants. * *p* < 0.05. Absolute critical values of correlation coefficients for 28 degrees of freedom: r_0.05_ = 0.36, r_0.01_ = 0.46, and r_0.001_ = 0.57.

A very large number of significant correlation coefficients were observed between hormone contents in cell sap and gene expressions (Figure 4). Statistically significant correlations (at the level of 0.001) were noted for 79 pairs (Figure 4). A positive correlation was observed for 66 pairs: 12-oxo-PDA–GA9, 12-oxo-PDA–GA6, 12-oxo-PDA–IAA sap, JA–GA19, JA–IAA-Glu, JA–ABA sap, JA–SA sap, JA–*BnWRKY57*, SA–GA9, SA–GA53, SA–GA6, SA–t-ZEA, GA15–GA6, GA9–GA7, GA9–GA3, GA9–GA6, GA9–IAAsp, GA9–*BnAOS*, GA9–*BnRGA*, GA53–GA1, GA53–t-ZEA, GA53–JA sap, GA44–c-ZEA-R, GA19–GA7, GA19–GA3, GA19–IAA-Glu, GA19–SA sap, GA19–*BnWRKY57*, GA20–GA5, GA20–c-ZEA, GA20–*BnICS1*, GA20–*BnABF2*, GA20–*BnARR6*, GA4–c-ZEA-R, GA7–GA3, GA7–GA1, GA7–IAAsp, GA7–IAA-Glu, GA7–SA sap, GA3–IAAsp, GA3–IAA-Glu, GA3–SA sap, GA6–t-ZEA, GA6–*BnAOS*, GA6–*BnRGA*, GA1–c-ZEA-R, GA5–c-ZEA, GA5–*BnICS1*, GA5–*BnABF2*, GA5–*BnARR6*, IAM–IAN, IAAsp–SA sap, IAAsp–*BnRGA*, IAA-Glu–ABA sap, IAA-Glu–SA sap, IAA-Glu–*BnWRKY57*, t-ZEA–JA sap, c-ZEA–*BnICS1*, c-ZEA–*BnABF2*, c-ZEA–*BnARR6*, ABA sap–SA sap, ABA sap–*BnWRKY57*, SA sap–*BnWRKY57*, *BnICS1*–*BnABF2*, *BnICS1*–*BnARR6,* and *BnABF2*–*BnARR6* (Figure 4). However, a strong negative correlation was observed for 13 pairs of traits: 12-oxo-PDA–ABA, 12-oxo-PDA–ABA sap, ABA–GA6, JA–JA sap, GA53–IPA, GA19–JA sap, GA6–ABA sap, IAM–IPA, IAN–IPA, IAA-Glu–JA sap, IPA–IAA sap, ABA sap–JA sap, and SA sap–JA sap (Figure 4).

**Figure 5:**
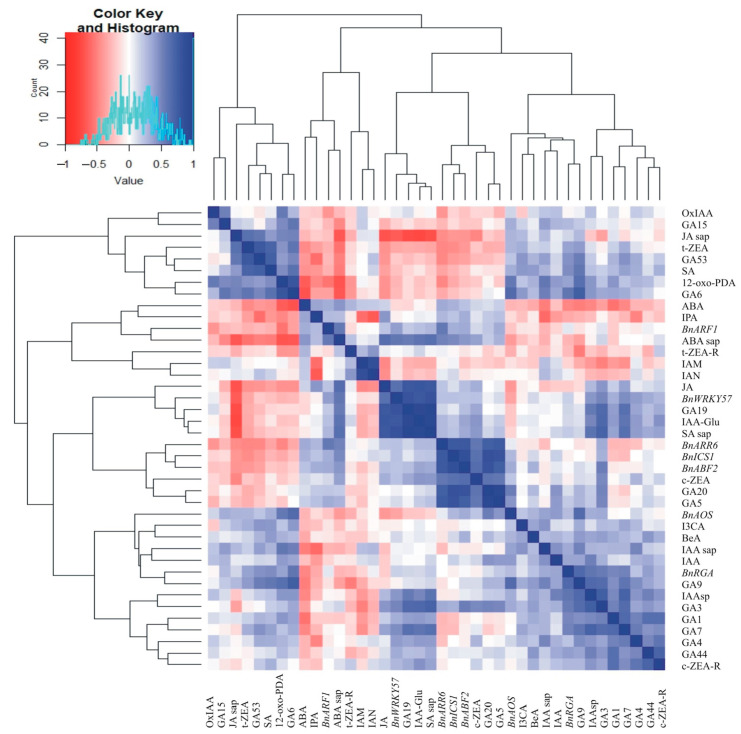
Heatmap showing hierarchical clustering, distinguishing four groups of hormone concentrations and gene expressions of oilseed rape plants.

The clustering performed using the unweighted pair group method with arithmetic mean (UPGMA) allowed us to distinguish four separate groups of features concerning hormone contents in juice and gene expression (Figure 5). One group consisted of eight traits: OxIAA, GA15, JA sap, t-ZEA, GA53, SA, 12-oxo-PDA, and GA6. The second group contained seven traits (ABA, IPA, *BnARF1*, ABA sap, t-ZEA-R, IAM, and IAN), and the third group contained eleven traits (JA, *BnWRKY57*, GA19, IAA-Glu, SA sap, *BnARR6*, *BnICS1*, *BnABF2*, c-ZEA, GA20, and GA5). In the fourth group, the remaining 14 traits were observed: *BnAOS*, I3CA, BeA, IAA sap, IAA, *BnRGA*, GA9, IAAsp, GA3, GA1, GA7, GA4, GA44, and c-ZEA-R (Figure 5).

## 3. Discussion

The processes of cold acclimation (CA) and deacclimation (DA) involve dynamic changes in the levels of stress-related hormones, including abscisic acid (ABA), jasmonic acid (JA), and salicylic acid (SA), which play crucial roles in regulating plants’ adaptive responses to low temperatures and the resumption of growth-related metabolism during a warm period.

Temperature is one of the factors that influences the concentration of hormones in plant tissues. ABA plays a crucial role in plant responses to cold stress by modulating the physiological and molecular pathways associated with cold acclimation across diverse species [52,53]. During cold acclimation, an increase in ABA levels is commonly observed, which is associated with the induction of cold tolerance genes and the accumulation of osmoprotectants and protective proteins, which increase the plant’s frost tolerance [54,55]. In our experiment, after three weeks of cold acclimation at 4 °C, the ABA content increased over 2-fold in both of the tested cultivars of winter oilseed rape (Table 1). These results were not surprising and were consistent with our previous studies conducted on other cultivars [10]. Moreover, an increased content of ABA has been detected during the early stages of seedling development in oilseed rape plants that had undergone pre-hardening [22]. A similar increase in ABA during CA has been reported in winter wheat, where it correlates with an improved freezing tolerance and increased sugar accumulation [8]. Furthermore, we also observed the increase in ABA concentration in cell sap during cold acclimation (Figure 2).

In our studies, during deacclimation, ABA content decreased in oilseed rape leaves, which is also confirmed by our previous research [10]. However, it increased in cell sap, especially in the cv. Kuga. This situation may be due to the redistribution of ABA in the plant. During DA, when growth resumes, ABA synthesized in the leaves during cold acclimation may be mobilized and transported to other parts of the plant [56]. This can lead to a decreased ABA concentration in leaf tissues while increasing its presence in the cell sap during moves through the vascular system to growth-active zones. Furthermore, ABA conjugates can be converted into free ABA in the sap, leading to an increase in its content [57]. Since the increase in ABA content is directly correlated with the increase in frost tolerance, it can be said that the decrease in ABA concentration during DA is also related to the decrease in frost tolerance, which we showed in previous studies [7].

The accumulation of ABA during cold exposure is correlated with the induction of cold-responsive (*COR*) genes and the activation of ABA-dependent transcription factors that mediate cold tolerance mechanisms [58]. A key transcription factor in this pathway is *ABF2* (ABA-responsive element-binding factor 2). The expression of *ABF2* increases in response to elevated ABA levels under cold conditions, enabling the activation of gene networks that promote osmolyte accumulation, antioxidative defence, and protective protein synthesis, all of which contribute to enhanced cold and frost tolerance [59], which is confirmed by our research. However, during deacclimation, we observed an even higher increase in the relative expression of *BnABF2*. Since *ABF2* can be regulated not only by ABA content but also by other stress-related molecules—ROS or sugars—it is possible that upregulation of the *ABF2* helps maintain cellular readiness for rapid reacclimation if temperatures drop again after premature deacclimation, facilitating the faster reactivation of cold-protective genes upon frost occurrence [60].

Jasmonic acid (JA), its precursors, and derivatives are one of the key regulators of plant responses to abiotic stresses, including cold stress, and play a significant role in acquiring plant tolerance to frost. Numerous studies have reported that JA content usually increases during cold acclimation in various plant species [8,61,62]. This is in agreement with our studies, where the leaf content of JA increased after cold acclimation. Furthermore, decreased precursor content (12-oxo-PDA) resulted in higher JA content and vice versa. The JA content after deacclimation depended on the cultivar—it increased in the cv. Kuga and decreased in the cv. Thure (Table 1).

A decreased JA concentration has been observed in cell sap during cold acclimation, with further reductions during deacclimation (Figure 2), which may be related to the formation of JA conjugates, which reduce detectable levels of free JA [63]. During deacclimation, further declines in JA content in cell sap may reflect a metabolic shift toward growth resumption, prioritizing gibberellins and auxin-mediated processes while downregulating JA-related defences and stress signalling [44]. Furthermore, energy and carbon demands during growth reactivation may lead to a diversion of linolenic acid and related precursors from JA synthesis towards membrane lipid remodelling, facilitating the adaptation of membrane fluidity to warmer temperatures [64].

The relative expression of *BnAOS* decreased during cold acclimation, which may be related to the plant’s transition from the initial cold stress signalling towards the stabilization phase of cold acclimation, where the emphasis shifts towards osmoprotection and membrane stabilization rather than active JA-mediated stress signalling [65]. Interestingly, during deacclimation, the expression pattern of *BnAOS* appears to be cultivar dependent (Figure 3).

Salicylic acid (SA) is another key phytohormone involved in the regulation of plant responses to abiotic stresses, including cold and freezing stress. During cold acclimation, increased SA accumulation has been reported in many species [8,66,67]. This SA accumulation during the initial phase of cold exposure is associated with the induction of cold-responsive genes, increased antioxidant capacity, and stabilization of the photosynthetic apparatus, which together contribute to improved cold and frost tolerance [66,67]. However, in our study, SA content during cold acclimation was lower than in non-acclimated plants (Table 1). Analogous results were obtained in other cultivars [10]. During deacclimation, the SA content increased slightly in both studied cultivars. The same profile of changes was observed in cell sap (Figure 2). This may be related to temporarily reduced or maintained basal SA synthesis to avoid crosstalk conflicts with ABA and JA pathways, which are crucial for low-temperature acclimation [67]. Typically, the relative expression of *BnICS1* increases during cold acclimation, which is confirmed by our studies. It is usually associated with increased antioxidant enzyme activity and reduced ROS accumulation, which may play a protective role at low temperatures [65,67]. However, during deacclimation, *BnICS1* expression patterns may differ among species, cultivars, and developmental stages, which was also observed in the current study (variety-dependent differences). Upregulation of *BnICS1* may reflect the readiness to rapidly reinduce cold-protective mechanisms if temperatures drop again after premature deacclimation.

The link between SA and frost tolerance is evident in studies showing that SA pretreatment or genotypes with higher endogenous SA levels exhibit enhanced frost tolerance due to reduced electrolyte leakage, higher photosynthetic activity, and stronger antioxidant defence under freezing stress [67,68]. Furthermore, SA may interact with ABA, JA, and ethylene signalling networks, allowing plants to fine-tune their response to cold stress [68].

Gibberellins (GAs) are key regulators of plant growth and development, and their signalling interacts with cold acclimation pathways to modulate frost tolerance. Cold acclimation typically leads to a decrease in bioactive GA content in leaves, contributing to the suppression of cell division and elongation, thereby conserving resources for cold protection [8,68,69]. In our study, all active gibberellins decreased their concentration during cold acclimation, which was accompanied by a decrease in the content of precursors (Table 1), which is a strategic adaptation to suppress growth and reallocate energy toward cold protection mechanisms. This decline stabilizes DELLA proteins, key repressors of GA signalling, which inhibit cell elongation and expansion, reducing energy consumption during cold conditions while facilitating the CBF/DREB-mediated cold-responsive (COR) gene expression [70].

During deacclimation, gibberellins content generally increases, reactivating growth and developmental transitions previously suppressed during periods of cold in order to conserve energy and increase frost tolerance. Our studies confirm this relationship, as all active forms of GAs increased their content in oilseed rape leaves during deacclimation (Table 1).

RGA encodes the DELLA protein, which inhibits gibberellin (GA) signalling, thereby limiting growth and increasing stress tolerance under unfavourable conditions. In *Brassica napus*, we noted a decrease in the relative expression of *BnRGA* during cold acclimation (Figure 3), which may seem contradictory to the stabilization of DELLA observed under cold stress conditions. An explanation for this phenomenon could be that cold-induced DELLA accumulation is mainly regulated at the protein stability level, not at the transcript level. Moreover, reduced GA concentrations during cold stabilizes DELLA proteins even if the RGA decreases [69]. Therefore, reduced *BnRGA* activity does not imply lower DELLA activity during cold but may reflect an adaptation to maintain a balance between growth restriction but not excessive inhibition. Moreover, the regulation of the GA pathway during cold acclimation is cultivar dependent [10]. It can be said that the variability of *BnRGA* expression reflected the changes in GA content during cold acclimation and deacclimation.

Auxins are another group of plant hormones that are regulators of plant growth and development, but their content could also affect frost tolerance during cold acclimation and deacclimation. Many studies report a decrease in free IAA content in leaves during cold acclimation, which is thought to slow down growth processes and redirect metabolic energy toward cold-protective mechanisms [71]. For example, in *Arabidopsis thaliana*, IAA levels decrease under cold stress, which correlates with reduced cell expansion and metabolic activity, thereby minimizing energy consumption while the plant builds osmoprotective and antioxidant capacities [72]. In our studies, during cold acclimation, we generally observed a decrease in the content of active auxin forms (IAA, IAA-sp, IAA-Glu, and OxIAA); however, the differences between cultivars were also visible (Table 1). Interestingly, the content of I3CA decreased to zero. Analogous decreases in IAA content during cold were observed in the cell sap of both cultivars (Figure 2). During deacclimation, IAA levels in leaves often increase, reactivating cell division and expansion with the increasing temperature, which is broadly confirmed by our study (Table 1).

The dynamics of IAA changes in cell sap may be variable. It could be constant or only slightly increased, reflecting a gradual transition towards growth resumption while maintaining readiness for potential reacclimation in the event of repeated frosts. This is also reflected in our study, as IAA content in the cell sap of cv. Kuga remained stable, while for cv. Thure, it increased statistically.

Auxin response factors (*ARFs*) are key transcriptional regulators [28]. Cold stress generally downregulates *ARF* expression, consistent with reduced auxin transport and signalling, to limit growth and shift plant metabolism toward survival [72]. This downregulation may further facilitate *CBF*/*DREB* and *COR* gene expression by minimizing the antagonistic effects of auxins on cold-responsive pathways [73]. However, *ARF* expression may increase under cold stress to regulate specific target genes involved in stress adaptation [74]. This was also shown in our study, where an increase in *BnARF1* expression was observed in cold-acclimated plants compared to non-acclimated plants in both cultivars. During deacclimation, *ARF* expression is reactivated, supporting the reinitiation of auxin-mediated growth processes, which was confirmed by our studies.

Cytokinins (CK) are regulators of plant growth, cell division, and developmental transitions, and their dynamics during cold acclimation and deacclimation are increasingly recognized as important in acquiring/losing frost tolerance. During cold acclimation, many species show a reduction in bioactive CKs, which is associated with growth inhibition [75]. In our study, no clear trend in the change in the content of active CKs could be found. During cold acclimation, their content either decreased (t-ZEA, c-ZEA-R) or increased (c-ZEA, t-ZEA-R). It is worth mentioning that c-ZEA is often regarded as a low-activity cytokinin. Although it is less active than its trans isomer, it is widely present in plants. Recent studies indicate that cis-zeatin also belongs to crucial regulators of plant growth and development [76]. Still, cold exposure usually causes a decrease in the content of CKs [77]. During deacclimation, reactivation of growth processes requires the restoration of CKs content to allow cell division and meristem reactivation. Our results show that the total content of the measured CKs increases in both studied cultivars (Table 1).

*ARRs* act as central components of CK signalling pathways, but their relative expression during cold acclimation may show different patterns depending on the isoform, tissue type, and cultivar. In oilseed rape, we observed that *BnARR* expression increased during cold acclimation and, depending on the cultivar, remained constant (cv. Kuga) or increased (cv. Thure) during deacclimation (Figure 3), reflecting the reactivation of CK signalling pathways to support growth resumption. However, this increase may vary among cultivars, suggesting genotype-dependent differences in the balance between growth reactivation and maintenance of cold tolerance. From a frost tolerance perspective, the reduction in CKs during cold acclimation is beneficial because it prioritizes stress responses over growth, whereas the careful restoration of CK signalling during deacclimation is necessary to reactivate growth. Additionally, it has been shown that the exogenous application of CKs improves the freezing tolerance of *Arabidopsis* seedlings [36].

Many correlations were detected between hormones and hormone-related genes (Figure 4). Those results reflect the occurrence of, among others, shared biosynthetic pathways, as many GAs correlate positively between themselves (e.g., GA15–GA6, GA9–GA7, GA9–GA3, and GA9–GA6). Negative correlations reflect antagonistic interactions (e.g., between ABA and growth-promoting gibberellins). The clustering analysis of hormone concentrations and hormone-related gene expressions revealed four groups (Figure 5) that may possibly present similar dynamics of change or possible crosstalk between particular components of the group upon deacclimation. For example, the fourth group that consists of *BnAOS*, I3CA, BeA, IAA sap, IAA, *BnRGA*, GA9, IAAsp, GA3, GA1, GA7, GA4, GA44, and c-ZEA-R portends to be connected to growth and development due to numerous auxins and gibberellins. Cold acclimation is regulated at various levels, making it difficult to identify a single key component, whether it be a specific phytohormone or gene. Our research illustrates the complex balance involved in this process. Better understanding the mechanisms that play a role in cold acclimation processes may help us to develop crop plants with higher levels of cold tolerance.

## 4. Materials and Methods

### 4.1. Plant Naterial

The experiment was conducted on winter oilseed rape (*Brassica napus* ssp. *oleifera* L.) on two cultivars, Kuga and Thure, which are cultivated in Poland. Both cultivars are a hybrid cultivar (F1).

According to COBORU (Development of Polish Official Variety Testing), the cv. Thure is a semi-dwarf cultivar, whose fully developed plants reach a height of about 126 cm tall, while the cv. Kuga plants can reach a height of about 143 cm.

In a previous experiment, their frost tolerance was characterized [7]. Plants that were cold-acclimated were characterized by a much higher tolerance to frost compared to non-acclimated plants. The frost tolerance of deacclimated plants decreased, but not to the level of basal frost tolerance noted for non-acclimated plants.

There were no differences between both cultivars. The temperature that was required to reduce regrowth by 50% (RT50) for the non-acclimated plants was about −4 °C, for the cold-acclimated plants about −13 °C, and for the deacclimated plants about −8 °C.

The seeds of both cultivars were obtained from Rapool, Poland.

### 4.2. Experimental Design and Sampling

**Figure 6:**
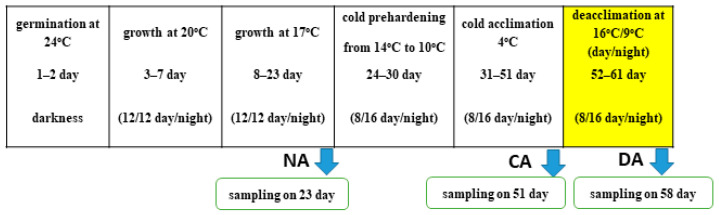
Experimental design.

The experimental design was similar to the previous model described in detail in [7]. Briefly, the fifty seeds were sown in Petri dishes (10 cm diameter) on moist filter paper for germination in the dark (24 °C, two days). The seedlings were transplanted to 11 pots (40 cm × 15 cm × 15 cm; 15 plants/pot) with a prepared soil mixture: the universal soil “Eco-Ziem Universal soil” (Eko-Ziem s.c., Jurków, Poland), pH = 5.5–7, sand, and the soil (degraded chernozem, made of loess, soil quality class I) from the cultivation plots of the University of Agriculture (Kraków—50°04′10″ N, 19°50′ 44″ E) (2:1:1). The plants were placed in growth chambers under controlled conditions. The pots in the growth chamber were rotated every day. The light intensity was the same during the entire experiment. The light was provided by LED lamps—type HORTI A (PERFAND LED, Trzebnica, Poland), modified to emit of constant intensity 350 μmol m^−1^·s^−1^. The relative humidity was 60 ± 5% during growth and deacclimation process and 40 ± 5% during cold acclimation, and the CO_2_ concentration was 380 ± 30 ppm. The experiment was performed in autumn/wintertime. The experimental design is presented in Figure 6.

The samples for all of the analyses were taken from the best-developed leaves in the rosette (not too young or senescing) of the non-acclimated plants (NA), cold-acclimated plants (CA), and deacclimated plants (DA).

The experimental model was designed to simulate the natural environmental conditions experienced by winter oilseed rape, which first grows under moderate temperatures (NA), then enters a cold period during autumn and winter (CA), followed by a sudden warming period (DA). Due to the cold-induced growth inhibition, plants remained in the rosette stage throughout the experiment, minimizing developmental variation between the NA, CA, and DA sampling points. This approach reflects the seasonal changes in the field.

The following analyses were made: the accumulation of phytohormones in leaves and cell sap and the expression of genes were connected with hormonal management.

### 4.3. Analysis of the Plant Hormones and Related Metabolites in Leaves and in Cell Sap

The collected leaves samples were frozen in liquid N_2_ and stored at −80 °C, then lyophilized and pulverized in a mixing mill (MM 400, Retsch, Kroll, Germany). Phytohormones were extracted from the dry leaves tissue (ca. 10 mg) according to [78]. Samples were spiked with stable isotope labelled internal standards and extracted in 1 mL of the methanol/water/formic acid mixture (MeOH/H_2_O/HCOOH, 15/4/1 *v*/*v*/*v*) [79]. Extraction was performed twice, and combined extracts were evaporated to dryness under nitrogen. The residues were dissolved in 1 mL of 3% MeOH in a 1 M HCOOH and purified on SPE cartridges (BondElut Plexa PCX, 30 mg, 1 mm, Agilent, Santa Clara, CA, USA). The phytohormone-containing fractions were eluted according to [10], evaporated to dryness under nitrogen, and reconstituted in 70 μL of acetonitrile (ACN).

To obtain cell sap, a mixture of a few fragments of leaves collected from different plants was pressed in a syringe with a filter disc using a hydraulic press. The whole amount of pressed sap was collected into the Eppendorf tube. The liquid samples (1 cm^3^) were acidified with formic acid to a final concentration of HCOOH 1 mol/dm^3^. An internal isotopic standard mixture consisting of deuterated IAA, ABA, SA, and JA labelled with nitrogen 15 N was added to each. Samples were purified on SPE cartridges Oasis MCX (Waters, Milford, MA, USA) and eluted by pure methanol. Each sample was evaporated to dryness and reconstituted in 100 μL methanol [80].

To perform the analysis, ultra-high performance liquid chromatography (UHPLC) was used. The UHPLC system (Agilent Infinity 1260, Agilent, Woldbrom, Germany) was coupled to a triple quadruple mass spectrometer MS/MS (6410 Triple Quad LC/MS, Agilent, Savage, MD, USA) with electrospray ionization (ESI). The samples were separated on an Ascentis Express RP-Amide analytical column (2.7 µm, 2.1 mm × 150 mm; Supelco, Bellefonte, PA, USA) at a linear gradient of water vs. ACN, both with 0.01% HCOOH. Multiple reactions monitoring (MRM) transitions were used to identify and quantify all compounds of interest (Supplementary Table S1). Quantitation was based on the calibration curves of pure standards of all compounds, taking into account the recovery of stable isotope labelled ISTDs. All standards were purchased from OlChemim (Olomouc, Czech Republic) at the highest available purity, whereas all solvents were of HPLC grade and were purchased from Sigma-Aldrich (Sigma-Aldrich, Darmstadt, Germany). Analyses were made in five biological replicates. Each biological replicate was performed in two technical replicates.

### 4.4. Accumulation of the BnRGA, BnARF1, BnARR6, BnABF2, BnISC1, BnAOS, and BnWRKY57 Transcripts: RNA Isolation, cDNA Synthesis, and qRT-PCR Reaction

The collected leaves samples (approximately 0.05 g) were frozen in liquid N_2_ and stored at −80 °C. RNA was extracted using the RNeasy Plant Mini Kit (Qiagen, Hilden, Germany) according to the manufacturer’s protocol. The quantity and purity of the RNA were checked using a UV-Vis Spectrophotometer Q5000 (Quawell, San Jose, CA, USA). Approximately 800 ng of RNA was subjected to genomic DNA elimination and reverse transcription using the QuantiTect Reverse Transcription Kit (Qiagen, Hilden, Germany). QuantStudio 3 Real-Time PCR System (Thermo Fisher Scientific, Waltham, MA, USA) was used for the RT-qPCR analysis. Primer sequences and sequence origins used in the study are given in Supplementary Table S2 and in the literature [81,82,83,84]. PCR reactions were run in triplicate in 96-well plates containing 15 µL of PowerUp SYBR Green PCR Master Mix (Thermo Fisher Scientific, Waltham, MA, USA), 800 nM of each primer, and approx. 30 ng template of cDNA. PCR amplification followed a protocol: 10 min at 95 °C, 40 cycles of 15 s at 95 °C, and 1 min at 60 °C. After PCR, a dissociation step was added to confirm the specificity of the reactions (15 s at 95 °C, 1 h at 60 °C, and 15 s at 95 °C). Row PCR data were analyzed using QuantStudio Design and Analysis v.1.5.0 software (Thermo Fisher Scientific, Waltham, MA, USA) and the relative standard curve method (Applied Biosystems), with *actin* as an endogenous control gene. The final results are presented as means of five biological replicates, which represent five leaves, each in three PCR technical replicates.

### 4.5. Statistical Analyses

The normality of the distribution of the observed traits (content of hormones) was tested using Shapiro–Wilk’s normality test [85]. Two-way analyses of variance (ANOVA) were carried out to determine the effects of the cultivar and treatment as well as cultivar x treatment interactions on the variability of the observed traits. The mean values and standard deviations of traits were calculated. Fisher’s least significant differences (LSDs) were estimated at the 0.05 significance level for cultivars and treatments as well as combinations of cultivars and treatments. Homogeneous groups were designated based on these LSD values. The concentrations of hormones in leaves were grouped using the unweighted pair group method with arithmetic mean (UPGMA) (Table 1). The relationships between observed traits were estimated using Pearson’s linear correlation coefficients and are presented in a heatmap. All analyses were performed using Genstat v. 23.1 statistical software [VSN International Genstat for Windows, 23 rd ed. VSN International: Hemel Hempstead, UK, 2023.]. Additionally, the content of hormones in cell sap and relative gene expressions were analyzed using Statistica 13.3 software (StatSoft, Tulsa, OK, USA), with the multifactorial analysis of variance (ANOVA) and Duncan’s test at a significance level of *p* ≤ 0.05. Values that are marked with the same letters did not significantly differ according to the Duncan test (*p* ≤ 0.05). The mean values together with the standard deviations are presented in the figures. Information about repetitions in a particular analysis is given in detail in chapters with descriptions of the respective methods.

## 5. Conclusions

Deacclimation in oilseed rape leads to a decrease in stress hormones such as abscisic acid and an increase in growth-promoting hormones, particularly gibberellins, reflecting a shift toward resumed growth under warmer conditions. Alterations in gene expression during deacclimation, including changes in, for example, *BnABF2* and *BnICS1*, may act as protective mechanisms to maintain or regain frost tolerance in the process of reacclimation if temperatures drop after a warm break. Differences were observed between hormone concentrations in deacclimated leaves and cell sap, for example, by differences in abscisic acid levels. Moreover, certain genes, such as *BnARF*, are characterized by the upregulated expression in deacclimated plants, which potentially supports the resumption of growth processes. These findings highlight the complex hormonal and molecular basics underlying plant responses to the deacclimation process.

## Figures and Tables

**Table 1:** The content [μg·g^−1^_DW_] of hormones in leaves of oilseed rape cv. Kuga and cv. Thure.

			Kuga			Thure					Cultivar Mean				Treatment Mean				
			NA	CA	DA	NA	CA	DA	LSD_0.05_	*F*	Kuga	Thure	LSD_0.05_	*F*	NA	CA	DA	LSD_0.05_	*F*
stress hormones—precursors	12-oxo-PDA	Mean	721	306	336	820	210	409	nsd	0.12	454	480	nsd	0.54	770X	258Z	372Y	101	<0.01
		s.d.	55	57	73	115	50	211			204	294			100	72	153		
	BeA	Mean	17040	14571	15684	15407	15276	16390	nsd	0.09	15765	15691	nsd	0.88	16223	14923	16037	nsd	0.08
		s.d.	1060	1113	1532	1185	397	1997			1561	1360			1365	871	1718		
	sum	Mean	17761	14878	16019	16227	15486	16798	nsd	0.12	16219	16170	nsd	0.92	16994X	15182Y	16409XY	1233	0.02
		s.d.	1104	1087	1505	1197	426	2103			1685	1426			1354	842	1772		
stress hormones—active forms	ABA	Mean	183	432	294	191	446	362	nsd	0.73	303	333	nsd	0.38	187Z	439X	328Y	85	<0.01
		s.d.	24	54	129	38	172	2			130	145			31	120	93		
	JA	Mean	507c	519c	3259a	685c	1763b	1421b	490	<0.01	1428	1290	nsd	0.32	596Z	1141Y	2340X	347	<0.01
		s.d.	10	47	772	102	447	195			1402	537			116	721	1105		
	SA	Mean	5365a	4090b	4110b	4299b	3889b	4130b	483	0.01	4522A	4106B	279	0.01	4832X	3990Y	4120Y	342	<0.01
		s.d.	446	344	261	550	252	268			701	394			734	303	250		
	sum	Mean	6056b	5041c	7663a	5175c	6098b	5914b	679	<0.01	6253A	5729B	392	0.01	5615Y	5570Y	6788X	480	<0.01
		s.d.	466	339	910	493	341	322			1257	550			648	643	1124		
gibberelins—precursors	GA15	Mean	5.14	4.46	3.92	8.30	3.86	4.70	nsd	0.09	4.51	5.62	nsd	0.11	6.72X	4.16Y	4.31Y	1.71	0.01
		s.d.	2.32	1.29	0.34	2.74	0.98	2.21			1.52	2.79			2.92	1.12	1.54		
	GA9	Mean	87a	60d	72b	75c	60bc	79d	6.14	<0.01	73	72	nsd	0.35	81X	60Z	76Y	4.34	<0.01
		s.d.	5.59	4.83	4.62	2.52	5.61	4.36			12	9.37			7.54	4.94	5.73		
	GA53	Mean	111a	42b	27c	21cd	16d	20cd	8.34	<0.01	60A	19B	4.81	<0.01	66X	29Y	23Y	5.90	<0.01
		s.d.	6.59	8.31	8.67	2.29	6.36	3.38			39	4.66			47	15	7.06		
	GA44	Mean	62	58	63	62	58	62	nsd	0.99	61	60	nsd	0.75	62X	58Y	62X	3.43	0.04
		s.d.	3.19	3.86	2.20	5.31	4.64	1.75			3.54	4.32			4.13	4.03	1.92		
	GA19	Mean	86c	73c	504a	155b	71c	178b	36.87	<0.01	221A	135B	21.29	<0.01	121Y	72Z	341X	26.07	<0.01
		s.d.	14	11	36	29	11	47			208	56			42	10	176		
	GA20	Mean	39b	30b	30b	50b	33b	415a	27.35	<0.01	33B	166A	15.79	<0.01	44Y	31Y	222X	19.34	<0.01
		s.d.	5.59	0.29	5.57	8	6	50			6	184			8	4	206		
	sum	Mean	390	268	699	371	242	759	nsd	0.06	452	457	nsd	0.74	380Y	255Y	729X	38	<0.01
		s.d.	19	13	30	24	25	89			189	233			23	23	70		
gibberelins—active forms	GA4	Mean	74	66	78	67	48	66	nsd	0.54	73A	60B	8.08	0.004	71X	57Y	72X	10	0.01
		s.d.	6.36	5.97	12	12	15	9			9	15			10	14	12		
	GA7	Mean	114a	36 d	129a	65b	41cd	59.07bc	18.27	<0.01	93A	55B	10.55	<0.01	90X	39Y	94X	13	<0.01
		s.d.	13	4.69	11	20	14	16			43	19			31	10	39		
	GA3	Mean	2118	274	3086	1727	546	3098	nsd	0.10	1826	1790	nsd	0.77	1923Y	410Z	3092X	305	<0.01
		s.d.	223	47	542	193	80	516			1248	1120			284	156	499		
	GA6	Mean	152	56	69	140	51	98	nsd	0.07	92	96	nsd	0.58	146X	53Z	83Y	18	<0.01
		s.d.	23	9	10	37	13	10			46	43			30	11	18		
	GA1	Mean	41	36	41	40	37	38	nsd	0.16	40	38	nsd	0.14	41X	37Y	40X	2	0.001
		s.d.	2.86	1.76	1.98	3.17	2.05	0.38			3.20	2.39			2.91	1.83	2		
	GA5	Mean	38bc	36bc	43b	26c	36bc	168 a	16.27	<0.01	39B	77A	9.4	<0.01	32Y	36Y	106X	12	<0.01
		s.d.	13	7	8	2.63	11	23			9	69			11	8	68		
	sum	Mean	2538	504	3446	2065	759	3527	nsd	0.05	2163	2117	nsd	0.71	2302Y	632Z	3487X	306	<0.01
		s.d.	244	45	531	212	100	510			1311	1208			330	153	493		
auxins—precursors	IAM	Mean	142c	540a	115c	263b	81c	139c	94	<0.01	266A	161B	55	<0.01	202.3Y	310.2X	127.2Z	66.8	<0.01
		s.d.	43	144	29	66	37	49			217	92			82.9	261.5	40		
	IAN	Mean	1838b	3268a	1323cd	1390bc	894d	1649bc	451	<0.01	2143A	1311B	261	<0.01	1614Y	2081X	1486Y	319.06	0.002
		s.d.	134	744	223	59	157	261			950	364			256	1350	286		
	sum	Mean	1980b	3808a	1439cd	1653bc	974d	1788bc	511	<0.01	2409A	1472B	295	<0.01	1816Y	2391X	1613Y	361.54	<0.01
		s.d.	150	865	216	124	144	259			1155	407			215.7	1603.9	290.5		
auxin—active form	IAA	Mean	119ab	109bc	108bc	121ab	89c	130a	20.91	0.02	112	113	nsd	0.78	120X	99Y	119X	15	0.01
		s.d.	9	22	9	17	3.32	24			15	24			13	18	21		
auxins—inactivated	IAAsp	Mean	225	63	262	213	61	207	nsd	0.44	183	160	nsd	0.20	219X	62Y	234X	44	<0.01
		s.d.	66	15	53	42	17	63			101	84			52	15	62		
	OxIAA	Mean	23b	24b	25b	32a	22b	22b	5.39	0.01	24	25	nsd	0.50	27X	23Y	24XY	3.81	0.04
		s.d.	5.05	2.41	4.60	2.91	2.59	5.87			3.97	6.01			5.82	2.54	5.28		
	IAA-Glu	Mean	78c	67c	321a	65c	64c	152b	37.08	<0.01	155A	94B	21.41	<0.01	72Y	65Y	237X	26	<0.01
		s.d.	22	10	60	5.14	8.88	23			126	45			17	9.09	99		
	I3CA	Mean	4347	0	1307	304	0	1428	nsd	0.14	1884	577	nsd	0.17	2325	0	1367	nsd	0.15
		s.d.	6253	0	189	95	0	170			3839	644			4682	0	181		
	sum	Mean	4674	153	1914	613	146	1810	nsd	0.15	2247	856	nsd	0.15	2643	149	1862	nsd	0.10
		s.d.	6214	12	149	88	15	133			3840	730			4663	14	144		
cytokinins—precursor	IPA	Mean	83d	67d	192c	165c	407a	252b	41.73	<0.01	114B	275A	24.09	<0.01	124Y	237X	222X	30	<0.01
		s.d.	15	4.75	30	25	20	63			60	110			47	180	56		
cytokinins—active forms	t-ZEA	Mean	4.73a	0.94b	0.32b	1.18b	0.96b	0b	1.35	0.001	2A	0.71B	0.78	0.002	2.96X	0.95Y	0.16Y	0.96	<0.01
		s.d.	0.79	1.32	0.50	1.32	1.45	0			2.20	1.18			2.13	1.31	0.38		
	c-ZEA	Mean	1.11	1.41	2.16	1.04	1.24	2.88	nsd	0.38	1.56	1.72	nsd	0.57	1.07Y	1.32Y	2.52X	0.71	<0.01
		s.d.	0.24	0.59	0.49	1.04	1.10	0.77			0.63	1.25			0.71	0.83	0.72		
	t-ZEA-R	Mean	1.67	2.35	2.02	1.64	1.90	1.71	nsd	0.50	2.01	1.75	nsd	0.09	1.66	2.12	1.86	nsd	0.05
		s.d.	0.28	0.74	0.40	0.18	0.34	0.23			0.56	0.26			0.22	0.59	0.35		
	c-ZEA-R	Mean	11	11	12	11	11	11	nsd	0.99	11	11	nsd	0.07	11	11	11	nsd	0.11
		s.d.	0.72	0.62	0.42	0.62	0.81	0.24			0.61	0.61			0.67	0.71	0.40		
	sum	Mean	19	16	16	15	15	16	nsd	0.04	17A	15B	1.22	0.01	17	15	16	nsd	0.09
		s.d.	1.24	1.85	1	2.12	2.23	0.55			1.97	1.74			2.7	2	0.79		

nsd—no significant differences; a, b, c, d, A, B, X, Y, and Z—in the rows, different letters of different sizes indicate statistically significant differences in mean values.

## Data Availability

Data are contained within the article and Supplementary Materials.

## Supplementary Materials

The following supporting information can be downloaded at: https://www.mdpi.com/article/10.3390/ijms26157408/s1.
