# Supplementary material for: Hormonal Balance in Relation to Expression of Selected Genes Connected with Hormone Biosynthesis and Signalling—The Effect of Deacclimation Process in Oilseed Rape
Source: Int J Mol Sci. 2025 Aug 1;26(15):7408. doi: 10.3390/ijms26157408 (PMC12347709; doi:10.3390/ijms26157408)
Supplement: Supplementary file 1 [file ijms-26-07408-s001.zip › Table S1 - optimized MS parameters.pdf]

Table S1. Optimized mass spectrometry parameters for phytohormone quantitation. The following conditions were found optimal for the analysis: capillary voltage 4 kV, gas temperature 350 °C, gas flow 12 L/min and nebulizer pressure of 35 psi. The measurements were made by using multiple reaction monitoring (MRM) in positive polarity. MassHunter software was used to control the LC-MS/MS system and for data analysis. For MRM parameters MassHunter Optimizer was used. The quantities of the internal standards (ISTD) are given in parenthesis.

Compound		Type of ion	Quantifier transition (precursor/product ions)	Fragmentor voltage (V)	Collision energy (V)	MRM Start Time (min.)
t-ZEA		[M+H]+	220.2/136.3	85	9	1
DHZ-N15	ISTD (10 pmol)	$[M+H]^+$	226.2/152	124	18	1.5
c-ZEA		$[M+H]^+$	220.2/136.3	85	9	
oxIAA		$[M+H]^+$	192.2/146.1	54	9	4.0
IAM		$[M+H]^+$	175.1/130	66	17	
t-Z-R-D5	ISTD (10 pmol)	$[M+H]^+$	357.3/225.2	116	17	5.12
t-Z-R		$[M+H]^+$	352.2/220.3	120	9	
c-ZEA-R		$[M+H]^+$	352.2/220.3	120	9	
IAAsp		$[M+H]^+$	291.2/130.1	54	25	6.4
IP		$[M+H]^+$	204.1/148.3	90	9	
BA-D4	ISTD (500 pmol)	$[M+H]^+$	128.1/84.1	61	13	
BeA		$[M+H]^+$	123.1/79.1	56	13	
IAA-Glu		$[M+H]^+$	305.2/130.1	58	29	
GA_3		$[M-H2O+H]^+$	329.3/311.3	100	14	8.15
GA1-D2	ISTD (10 pmol)	$[M-H_2O+H]^+$	333.3/287.2	58	9	
GA1		[M-H2O+H]+	331.3/285.3	100	14	
I3CA		$[M+H]^+$	162.2/118.1	58	9	
IAA-D5	ISTD (100 pmol)	$[M+H]^+$	181.1/135.1	38	14	
IAA		$[M+H]^+$	176.1/130.3	51	9	
SA-D4	ISTD (500 pmol)	$[M+H]^+$	143.2/125.2	80	14	
SA		$[M+H]^+$	139.2/121.2	80	14	
GA6-D2	ISTD (10 pmol)	$[M-H_2O+H]^+$	331.3/115.1	96	5	10.4
GA_6		$[M-H2O+H]^+$	329.3/283.3	104	14	
IAN-D4	ISTD (100pmol)	$[M+H]^+$	161.1/134.1	66	13	12.0
IAN		$[M+H]^+$	157.1/130.1	71	13	
ABA-D6	ISTD (30 pmol)	[M-H2O+H]+	253.4/191.3	80	14	14.6
ABA		$[M-H2O+H]^+$	247.4/187.2	80	14	
GA5-D2	ISTD (10 pmol)	$[M-H_2O+H]^+$	287.3/115.0	96	5	15.45
GA5		$[M-H_2O+H]^+$	285.1/115.0	96	5	
GA_{20}		$[M-H_2O+H]^+$	287.3/115.0	96	5	
GA_{19}		$[M-H_2O+H]^+$	345.2/299.1	80	9	16.8

Compound		Type of ion	Quantifier transition (precursor/product ions)	Fragmentor voltage (V)	Collision energy (V)	MRM Start Time (min.)
t-ZEA		[M+H]+	220.2/136.3	85	9	1
GA_{44}		$[M+H]^+$	347.2/301.1	125	9	
JA-D5	ISTD (100 pmol)	$[M+H]^+$	216.3/153.2	80	5	19.5
JA		$[M+H]^+$	211.3/151.2	80	14	
GA_7		$[M-H_2O+H]^+$	313.2/223.1	104	14	18.5
GA4-D2	ISTD (10 pmol)	$[M-H_2O+H]^+$	317.3/271.2	88	9	
GA_4		$[M-H_2O+H]^+$	315.3/269.3	100	14	
GA_{53}		$[M-CO_2]^+$	303.2/285.1	100	9	
GA_9		$[M-H_2O+H]^+$	271.3/225.2	136	13	22.25
GA_{15}		$[M+H]^+$	331.2/285.1	115	9	
dinor-12-oxo-OPDA-D5	ISTD (10 pmol)	$[M+H]^+$	270.3/252.2	84	5	
12-oxo-PDA		$[M+H]^+$	293.3/275.2	68	9	24.54
