# Supplementary material for: Hormonal Balance in Relation to Expression of Selected Genes Connected with Hormone Biosynthesis and Signalling—The Effect of Deacclimation Process in Oilseed Rape
Source: Int J Mol Sci. 2025 Aug 1;26(15):7408. doi: 10.3390/ijms26157408 (PMC12347709; doi:10.3390/ijms26157408)
Supplement: Supplementary file 1 [file ijms-26-07408-s001.zip › Table S2 - primers for qPCR.pdf]

Supplementary Table 2. Genes, sequence origins and designed primers used in the study.

Gene name	Forward primer	Reverse primer	GenBank ID / Sequence origins
ABF2	TCTCAGAGTCAGAGGCAGCA	ACACTGGCGTAACAGGAGACA	HE616527.1
RGA	GCTGGTGGTTCCGATTACG	GACGCTTGGTGCTCTGACTG	Gao et al. 2012
ARR6	CGTTATTAGCGATGTCGACATG	CAATACGCACCGGTTTAAGTAG	Jiang et al. 2022
ICS1	CTTCCAGCTACAATCCCTGTC	TGGCGAGGAGAGTGAATTTG	Park et al. 2021
AOS	TGCAACAACCTCTCTCCTTC	GATATCAACCGCTTGCGACTA	XM_013888091.2
ARF1	CGAGAACGCTTACGACCATGTC	ACCGTTGCTCTCTTCGCCG	Wen et al. 2019
WRKY57	CCAAGGCTATGGATCCTCACA	GCCGCCTTAGCTCACTTGAGT	MG699908.1
Actin	TCAGTGGTGGTTCGACCATGT	CCGTGATCTCTTTGCTCATACG	AF111812.1

Jiang, J.-J.; Li, N.; Chen,W.-J.; Wang, Y.; Rong, H.; Xie, T.;Wang, Y.-P. Genome-Wide Analysis of the Type-B Authentic Response Regulator Gene Family in *Brassica napus*. Genes 2022, 13, 1449. https://doi.org/10.3390/genes13081449

Park, S.-H.; Lee, B.-R.; La, V.H.; Mamun, M.A.; Bae, D.-W.; Kim, T.-H. Drought Intensity-Responsive Salicylic Acid and Abscisic Acid Crosstalk with the Sugar Signaling and Metabolic Pathway in *Brassica napus*. Plants 2021, 10, 610. https://doi.org/10.3390/plants10030610

Gao, Y., Chen, J., Zhao, Y. et al. Molecular cloning and expression analysis of a RGA-like gene responsive to plant hormones in *Brassica napus* . Mol Biol Rep 39, 1957–1962 (2012). https://doi.org/10.1007/s11033-011-0943-7

Wen J, Guo P, Ke Y, Liu M, Li P, et al. (2019) The auxin response factor gene family in allopolyploid *Brassica napus*. PLOS ONE 14(4): e0214885. https://doi.org/10.1371/journal.pone.0214885
